# TIGIT Blockade Potentiates the Anti-Leukemic Activity of Exercise-Mobilized Donor Lymphocytes and Expanded γδ T-Cells

**DOI:** 10.3390/cancers18050797

**Published:** 2026-02-28

**Authors:** Grace M. McKenzie, Josie Voss, Emmanuel Katsanis, Richard J. Simpson, Forrest L. Baker

**Affiliations:** 1 School of Nutritional Sciences and Wellness, University of Arizona, Tucson, AZ 85721, USA; 2 Department of Medicine, University of Arizona, Tucson, AZ 85721, USA; 3 Department of Pediatrics, University of Arizona, Tucson, AZ 85721, USA; 4 University of Arizona Cancer Center, University of Arizona, Tucson, AZ 85721, USA; 5 Department of Immunobiology, University of Arizona, Tucson, AZ 85721, USA; 6 Department of Exercise Science, University of South Carolina, Columbia, SC 29208, USA

**Keywords:** gamma delta T-cells, checkpoint inhibitor, DLI, aminobisphosphonate, exercise, graft-versus-host disease, PD-1, CD8 T-cells, NK cells

## Abstract

**Background:** Donor lymphocyte infusion (DLI) is commonly used to prevent or treat leukemic relapse following allogeneic hematopoietic cell transplantation; however, efficacy is limited by immune exhaustion, checkpoint-mediated inhibition, and the risk of graft-versus-host disease (GvHD). Gamma delta (γδ) T-cells represent a promising “off-the-shelf” adoptive cell therapy (ACT) with favorable safety and MHC-independent cytotoxicity, yet their function is similarly constrained by the leukemic tumor microenvironment (TME). Acute exercise mobilizes cytotoxic lymphocyte subsets, and is an emerging strategy to enhance cellular immunotherapies, including DLI and expanded γδ T-cells. This study examined how exercise-mobilized lymphocytes and exercise-expanded γδ T-cells interact with TIGIT blockade to improve anti-leukemic activity. **Methods:** Healthy participants completed an acute cycling bout, after which peripheral blood mononuclear cells (PBMCs) and ex vivo expanded γδ T-cells were phenotyped and cytotoxicity was determined against leukemia cells with TIGIT checkpoint inhibition. The therapeutic relevance of combining TIGIT blockade with rest- or exercise-expanded γδ T-cells was further evaluated in NSG-IL15 mice challenged with K562-luc leukemia. **Results:** Acute exercise increased circulating CD8^+^ and γδ T-cells with higher TIGIT and PD-1 expression. Exercise-expanded γδ T-cells maintained increased PD-1 and TIGIT expression and exhibited increased co-expression of DNAM-1 and TIGIT. Exercise mobilized PBMCs and exercise-expanded γδ T-cells demonstrated enhanced cytotoxicity, further amplified by TIGIT blockade. In vivo, TIGIT-treated exercise-expanded γδ T-cells modestly improved tumor suppression and prolonged tumor-free survival compared to untreated controls. **Conclusions:** Exercise primes DLI and γδ T-cell products for enhanced responsiveness to TIGIT checkpoint inhibition. Targeting TIGIT likely augments DNAM-1 dependent cytotoxicity and improves anti-leukemic activity, supporting the integration of exercise-enhanced DLI and γδ T-cell therapies with immune checkpoint blockade as a safe strategy to improve relapse control in leukemia.

## Simple Summary

Donor lymphocyte infusions and gamma delta T-cells are types of adoptive cell therapy that help treat leukemic relapse after stem cell transplantation, but their activity is inhibited by the tumor microenvironment. Immune checkpoint inhibitors can restore immune function, yet their effectiveness in blood cancers has been modest. Additionally, exercise is an emerging physiological strategy to enhance the composition and function of therapeutic immune cells. This study investigated whether exercise could improve the anti-leukemic activity of donor lymphocytes and expanded gamma delta T-cells when combined with the immune checkpoint inhibitor, TIGIT. Herein we demonstrate that exercise primes donor lymphocytes and gamma delta T-cells for enhanced responsiveness to checkpoint inhibition, resulting in improved leukemia cell killing. These results highlight the integration of exercise as a safe adjunct to immune checkpoint therapy to optimize cellular immunotherapy for leukemia.

## 1. Introduction

Leukemic relapse following allogeneic hematopoietic cell transplantation (alloHCT) is a major cause of treatment failure [1,2]. Donor lymphocyte infusion (DLI) is widely used to prevent or treat post-transplant relapse by augmenting graft-versus-leukemia (GvL) activity [3]. While DLI is particularly effective in chronic myeloid leukemia (CML), its utility is limited by suboptimal remission rates and the risk of graft-versus host-disease (GvHD) [4]. Gamma delta (γδ) T-cells have emerged as a promising adoptive cell therapy (ACT) product for relapse control, as their capacity for MHC-independent cytotoxicity, unconventional recognition mechanisms, and proliferative capacity make them targets for “off-the-shelf” immunotherapy [5,6,7,8]. Importantly, γδ T-cell infiltration correlates with improved clinical outcomes in several hematological malignancies, including CML, highlighting the need to enhance their efficacy [9,10,11,12].

Despite therapeutic efficacy, both DLI and γδ T-cell products are limited by the tumor microenvironment (TME), which induces exhaustion and attenuates cytotoxicity and persistence [13]. The leukemic TME downregulates activating receptors, stress ligands, and cytotoxic mediators (i.e., perforin, granzyme-B) while overexpressing inhibitory ligands such as PD-L1, Galectin-9, and CD155, collectively suppressing T- and γδ T-cell function [14,15,16,17]. The DNAM-1/PVR (CD226/CD155) axis is a central pathway in γδ T-cell, NK-cell and CD8^+^ T-cell tumor recognition, but this axis can be disrupted through TIGIT binding of PVR [18,19,20]. Importantly, T-cell TIGIT is upregulated on exhausted γδ T-cells and lymphocytes from leukemia patients [21,22,23]. These suppressive features underscore the need for strategies that enhance both DLI-mediated GvL and γδ T-cell–based therapies.

Immune checkpoint inhibitors (ICIs) have revolutionized cancer therapy by reinvigorating exhausted T-cells and improving survival across multiple solid tumors [24,25,26]. However, efficacy in hematological malignancies is modest [27,28], hindered by low immunogenicity and the immunosuppressive TME enriched with MDSCs [29], regulatory T-cells [30], and inhibitory cytokines [31,32]. Consequently, ICIs are typically used in relapse or maintenance settings rather than in frontline therapy [33]. Given these constraints, combination therapies are increasingly necessary to bolster ICI responses. In leukemia patients, elevated TIGIT, TIM-3, and PD-1, expression on CD8^+^ T-cells, NK-cells, and γδ T-cells subsets suggests a specific vulnerability to checkpoint-mediated inhibition [21,34,35]. Thus, combining DLI products and γδ T-cells with ICIs offers a promising strategy to augment T-cells and generate a more robust ACT product.

Acute exercise provides an additional, biologically meaningful, tool for improving the composition and function of DLI and γδ T-cells products [18,36,37]. Exercise mobilizes NK-cells, γδ T-cells, and CD8^+^ T-cells into the circulation in a catecholamine-dependent manner, enhancing tumor immunosurveillance and cytotoxicity [38,39,40]. Exercise-mobilized peripheral blood mononuclear cells (PBMCs) are enriched with effector-memory and cytotoxic phenotypes, demonstrating superior anti-tumor activity relative to resting DLIs [41,42,43,44]. Similarly, exercise-expanded γδ T-cells exhibit greater proliferative capacity, phenotypic diversity, and cytotoxicity than standard-expanded counterparts [18,36,45,46]. Leveraging the unique advantages of exercise-mobilized DLIs (DLI-X) and exercise-expanded γδ T-cells may ameliorate the clinical limitations of DLIs and γδ T-cells in patients with hematological malignancies. However, evidence on how acute exercise regulates inhibitory checkpoint ligands on these cells remains limited.

This study investigated how exercise-mobilized DLIs and exercise-expanded γδ T-cell products interact with ICIs to enhance anti-leukemic activity in a CML model. We identified exercise-induced shifts in TIGIT, PD-1, LAG-3, and TIM-3 expression across cytotoxic lymphocyte subsets and expanded γδ T-cells, demonstrated that TIGIT blockade augments in vitro cytotoxicity, and observed modest improvements in tumor control, in vivo. These findings indicate that targeting TIGIT potentiates the anti-leukemic activity of exercise-expanded cells, highlighting a safe and promising combinational strategy to improve relapse control in leukemia.

## 2. Materials and Methods

### 2.1. Participants

Twenty-six healthy participants (nine females; age: 32.4 ± 9.9 years; height: 174.15 ± 13.7 cm, weight: 81.5 ± 28.4 kg; BMI: 27.1 ± 10.1 kg/m^2^; $\dot{V}$O_2_max 40 ± 9.97 mL/kg/min; resting HR 71.2 ± 15.3 bpm; resting Systolic BP 127.7 ± 15.2 mmHg; resting Diastolic BP 83.6 ± 11.2 mmHg; fasting Glucose 93.8 ± 9.6 mg/dL; fasting Cholesterol 131.9 ± 28.4 mg/dL) were enrolled in the registered clinical trial “Exercise as an Immune Adjuvant for Allogeneic Cell Therapies (Allo-X)” (NCT06643221). Participants were excluded if they used immune-modulating medications or tobacco within the past six months, had asthma, or presented with abnormal blood pressure, glucose, or lipid values. All individuals were classified as “low risk” for maximal exercise testing per American College of Sports Medicine (ACSM)/American Heart Association (AHA) criteria.

All participants completed two laboratory visits separated by one to three weeks. Participants were required to abstain from vigorous exercise for 24 h before each visit and to arrive at the laboratory following an overnight (8–12 h) fast, having consumed only water. This was confirmed verbally upon their arrival at the laboratory. All testing was performed at the University of Arizona College of Medicine between 07:00 and 10:00 am to minimize diurnal variation. Participants provided written informed consent and medical history after a thorough explanation of the procedures and risks. The study was conducted in accordance with the Declaration of Helsinki and approval from the Human Subjects Protection Program at the University of Arizona (Tucson, AZ; #1801161041), with written consent from all participants.

### 2.2. Exercise Protocol

Participants completed a maximal graded exercise test on a cycle ergometer (Excalibur Sport, Lode B.V., Groningen, The Netherlands) to determine maximal oxygen uptake ($\dot{V}$O_2_max). Heart rate and respiratory gas exchange were continuously recorded during the test (Quark CPET, COSMED, Pabona di Albona Laziale, Italy). After a 5 min warm-up at 50 watts (W), resistance increased by 15 W each minute until volitional exhaustion [47]. Cycling cadence was maintained at ≥60 rpm a rating of perceived exertion (RPE (Modified BORG scale); 0–10) was recorded during the final 15 s of each stage. Linear regression plots were produced for each participant to determine cycling powers corresponding to 50%, 60%, 70%, and 80% $\dot{V}$O_2_max for the subsequent laboratory visit.

On visit 2, subjects were asked to complete a 5 min warm-up followed by four consecutive 5-min stages at 50%, 60%, 70%, and 80% $\dot{V}$O_2_max. Heart rate, oxygen uptake, and RPE were recorded throughout the trial. An indwelling antecubital catheter (BD, Franklin Lakes, NJ, USA) was placed for blood collection at rest (REST) and during exercise (EX) at the 80% $\dot{V}$O_2_max intensity. Blood was drawn into acid-citrate dextrose ACD tubes for PBMC isolation and γδ T-cell expansion, and into EDTA tubes for whole blood phenotyping.

### 2.3. Blood Sample Analysis and Processing

**Figure 1:**
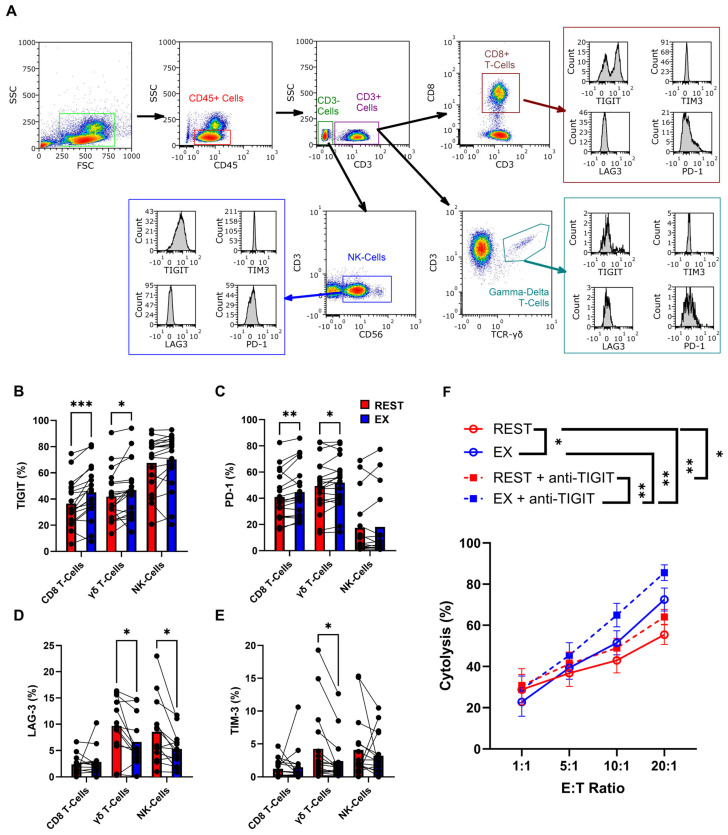
Acute exercise regulates immune checkpoint profiles in PBMC subsets and augments their anti-leukemic function under TIGIT blockade. (**A**) Gating strategy employed to determine immune cell phenotypes and immune checkpoint expression among them, within the CD45+ gate based on forward (FSC) and side scatter (SSC). (**B**–**E**) The percentage of immune checkpoint ((**B**) TIGIT, (**C**) PD-1, (**D**) LAG-3, (**E**) TIM-3) expression on leukocyte subsets (CD8^+^ T-cells, γδ T-cells, NK-cells) within isolated PBMCs from before (REST) and during (EX) exercise. (**F**) The in vitro anti-tumor activity of REST and EX PBMCs with or without anti-TIGIT against K562, assessed at 24 h via bioluminescent imaging (BLI). Data are represented as mean ± SEM; *, *p* < 0.05; **, *p* < 0.01; ***, *p* < 0.001; mixed effects one-way ANOVA with Fisher’s LSD *post hoc* test (**B**–**E**). Repeated measures two-way ANOVA or LMM with Tukey *post hoc* test (**F**).

Complete blood counts were immediately performed on whole blood samples treated with EDTA using a hematology analyzer (Beckman Coulter DxH 560 AL (Beckman Coulter, Indianapolis, IN, USA)). EDTA whole blood (100 µL) was labeled with FcR blocking reagent for 15 min (Miltenyi Biotec, San Diego, CA, USA), followed by respective antibodies (Table S1) and then incubated in the dark for 30 min, at room temperature. Blood samples were then mixed with a red blood cell lysing buffer (Miltenyi Biotec, San Diego, CA, USA) for 20 min, washed three times with phosphate-buffered saline (PBS), before being resuspended in 300 µL of PBS. The samples were then analyzed by flow cytometry (MACSQuant 16, Miltenyi Biotec, San Diego, CA, USA), and the percentage of each immune cell phenotype was multiplied by the lymphocyte or monocyte count from the automated hematology analyzer to determine the number of cells/µL of whole blood. Expression and intensity of population subtypes were determined within the CD45+ gate based on forward and side scatter using FlowLogic (Figure 1A) (Inivai Technologies, Mentone, VIC, Australia).

ACD tubes were used at blood collection, from which PBMCs from whole blood were isolated by density gradient centrifugation (Ficoll Paque Plus, Cytiva, Marlborough, MA, USA) and cryopreserved in liquid nitrogen at a concentration of 10 × 10^6^ cells/mL freezing media (90% FBS, 10% DMSO) until further use in in vitro or in vivo experiments.

### 2.4. γδ T-Cell Expansion

The γδ T-cell expansion protocol was performed as previously described [36]. Briefly, cryopreserved PBMCs were thawed and seeded at a concentration of 1 × 10^6^ cells/mL in a 24-well plate with culture media consisting of 500 IU/mL IL-2 and 5 μM/L of Zoledronic Acid (ZOL) (Sigma-Aldrich, St. Louis, MO, USA), in culture media (RPMI-1640 with 10% FBS and 1% penicillin-streptomycin (Sigma-Aldrich)) [48]. γδ T-cell cultures were changed every 3–4 days, with fresh culture media containing only IL-2 (500 IU/mL). Cells were harvested after 12–14 days to determine number and purity (Table S1). Expanded γδ T-cells were cryopreserved for further phenotypic and functional analysis (in vitro and in vivo).

### 2.5. Immune Checkpoint Expression

The expression of potential ICI targets (PD-1, TIGIT, LAG-3, TIM-3) were determined by flow cytometry on PBMCs and expanded γδ T-cells at manufacturer-recommended concentrations. Briefly, cryopreserved PBMCs or expanded γδ T-cells were thawed, washed, and counted by flow cytometry. The PBMCs or expanded γδ T-cells (3 × 10^5^ cells) were stained for 15 min with an FcR-blocking reagent (Miltenyi Biotec, San Diego, CA, USA), followed by antibodies (Table S1) for 30 min at 37 °C in the dark and then analyzed by 14-color flow cytometry (MACSQuant 16, Miltenyi Biotec: Bergisch Gladbach, Germany).

### 2.6. Cytotoxicity Assays

The in vitro cytotoxic function of exercise mobilized PBMCs and exercise-expanded γδ T-cells in combination with anti-TIGIT were assessed by bioluminescence (LagoX Spectral Instruments Imaging, Tucson, AZ, USA) cytotoxicity assays as previously described [49]. On the day of the cytotoxicity assay, PBMCs or expanded-γδ T-cells were thawed in a 37 °C water bath and stimulated with IL-2 (500 IU/mL) for 1 h to improve activation and recovery of γδ T-cell functions. To investigate TIGIT-mediated suppression of PBMC or expanded γδ T-cell cytotoxicity, effector cells populations were incubated with either culture media alone, an REA/IgG1 isotype control, or an anti-human TIGIT (Tiragolumab biosimilar) antibody (10 µg/mL, InVivoSIM, Bio X Cell, Lebanon, NH, USA) for 30 min prior to performing the cytotoxicity assay, as previously described [6]. K562 target cells were co-cultured with the restimulated, PBMCs or expanded γδ T-cells at 0:1 (control for spontaneous lysis of targets), and 1:1, 5:1, 10:1, and 20:1 γδ T-cell effector:target (E:T) ratios, all within a 96-well plate. PBMC and γδ T-cell cytotoxicity was determined at 24 h and 4 h, respectively, by the addition of D-Luciferin post-incubation.

### 2.7. Xenogeneic Mouse Experiments

**K562-luc2 and γδ T-cell preparation for injection**: The HLA-deficient CML cell line K562-luc2 (ATCC: CCL-243) was used to induce a widespread tumor in mice and used as the target cell in cytotoxicity assays and was maintained as previously described [41]. Briefly, K562-luc2 cells were thawed from cryopreservation 48 h before tumor injections and maintained at 37 °C, 5% CO_2_ in Iscove’s DMEM supplemented with 10% FBS and 8 µg/mL blasticidin for 2 days. Cryopreserved γδ T-cells from three donors were thawed and resuspended in culture medium supplemented with IL-2 (500 IU/mL) for one hour at 37 °C, 5% CO_2_ to improve activation and recovery of γδ T-cell functions before use in murine models.

**NSG IL-15 murine model:** All mouse experiments were completed in compliance with Institutional Animal Care and Use Committee (IACUC) guidelines at the University of Arizona under an approved protocol (#17-338). Eight- to twelve-week-old, male and female mice NOD.Cg-Prkdcscid Il2rgtm1Wjl Tg(IL15)1Sz/SzJ (NSG-Tg(Hu-IL15) obtained from Jackson Laboratory, were used for xenotransplantation of K562-luc2 tumor cells and expanded-γδ T-cells. On Day -1, mice were whole body irradiated with 100 cGy with a RadSource X-ray irradiator (RadSource: Brentwood, TN, USA) to enhance the engraftment of human cells. On Day 0, mice were injected intravenously through the lateral tail vein with 10 × 10^6^ rest- or exercise-expanded-γδ T-cells stained with anti-human TIGIT (10 ug/mL) (Tiragolumab Biosimilar) or human IgG1 isotype control (7.16 ug/µL) (In Vivo SIM, Bio X Cell, Catalog #BE0433, #BE0297, Lebanon, NH, USA). Four hours later, mice were intravenously injected with 5 × 10^5^ K562-luc2 cells. On Day +1 mice were intraperitoneally (IP) injected with ZOL (50 μg/kg) to sensitize the tumors to γδ T-cell-mediated lysis. Mice that did not receive the treatment conditions were instead injected with an equal amount of saline. Each expanded-γδ T-cell sample (REST or EX; with or without TIGIT) was injected into 2–3 mice, resulting 6–7 mice per group, and a total of 41 mice. Mice were group-housed (2–3 mice per cage) to diminish stress. An individual who was not involved in data collection or analysis performed the group allocation and the individual responsible for data collection and analysis was blinded to the group assignments. All mice were monitored daily until euthanized, via exposure to a lethal dose of CO_2_, following institutional guidelines and no mice were excluded from the analysis. This work has been reported in line with the ARRIVE guidelines 2.0.

**γδ T-cell engraftment:** Human γδ T-cells engraftment was determined on Day +14 of the experiment. A sample of blood (50–100 µL) was collected from the tail vein of each mouse. 25 µL of whole blood samples were stained and analyzed by flow cytometry using the following directly conjugated antibodies: CD3-FITC, CD45mouse-PE-Vio770, CD45human-APC, TCR-γδ PerCP-Vio700 (Miltenyi Biotec, Bergisch Gladbach, Germany). All antibodies, excluding CD45mouse, are reactive with human antigens. CD45mouse was used to exclude mouse leukocytes from analysis.

**Bioluminescence imaging (BLI):** All mice were imaged weekly to monitor tumor progression on the Lago-X Spectral Instruments bioluminescent imager. Briefly, mice were injected IP with D-luciferin, potassium salt reconstituted in Dulbecco’s phosphate-buffered saline (15 mg/mL, Gold Biotechnologies, St. Louis, MO, USA) at a concentration of 10 µL/g body weight (BW) and anesthetized with 2% isoflurane prior to imaging. Bioluminescent images were gathered by 5 min exposure unless saturation increased at which 3 min, 1 min, or 30 s exposures were performed.

**Morbidity/GvHD scoring**: Mice were weighed and scored for symptoms of xenogeneic GvHD based on the clinical parameters described previously [18]. Briefly, scores were assessed based on the following symptoms of clinical GvHD: skin integrity (0–2): 0 = normal, healthy skin; 1 = scaling of paws/tail; 2 = dehydrated, obvious areas of denuded skin; fur integrity (0–2): 0 = normal, fluffy, and elastic fur; 1 = mild to moderate ruffling; 2 = soiled, stiff, and rough fur; posture (0–2): 0 = normal posture; 1 = hunching only at rest; 2 = severe hunching, sunken or distended abdomen; activity (0–2): 0 = normal, responsive and vocal; 1 = mild to moderately decreased; 2 = unresponsive, separates from group, circling, head pressing, hind limb paralysis; weight loss (0–2): 0 = <10%; 1 = 10% to 20%; 2 = >20%; and diarrhea (0–1): 0 = no; 1 = yes. Clinical signs, or a combination of them, were considered a moribund condition, and thus, mice were sacrificed under the following condition(s): GvHD ≥ 6, ≥25% BW loss, hind limb paralysis, and/or impaired ambulation.

### 2.8. Statistical Analysis

Statistical analyses were completed using GraphPad Prism V10 (GraphPad Software, San Diego, CA, USA). Data are represented as mean ± SD unless otherwise stated. Paired *t* tests or Wilcoxon matched-pair signed rank test were used to quantify differences in the proportion, total number, and phenotypes of isolated PBMCs or expanded γδ T-cells before and during exercise. Linear mixed models (LMMs), with Bonferroni or Tukey correction for multiple comparisons, were built to detect main effects of group (REST vs. EX), dose ratios (E:T), condition (TIGIT antibody blockade), treatment (ZOL sensitization), and interaction effects for in vitro cytotoxic function of the exercise-expanded γδ T-cells. Multiple LMMs with Bonferroni correction for multiple comparisons were used to detect main effects of group (REST vs. EX vs. tumor control), treatment (TIGIT antibody blockade), and multiple interaction effects for leukemic burden (BLI) and BW. Simple survival analysis (Kaplan–Meier) was used to detect differences in overall survival and tumor-free survival. Significance was accepted at *p* < 0.05.

## 3. Results

### 3.1. Exercise Alters Immune Checkpoint Expression in PBMC Subsets and Enhances Their Anti-Leukemic Activity with TIGIT Blockade

An acute bout of cycling significantly increased circulating CD4^+^ T-cells, CD8^+^ T-cells and γδ T-cells (Table 1). Using a comprehensive immunophenotyping panel, we assessed exercise-induced changes in the expression of four common immune checkpoint receptors across major leukocyte subsets. Among cytotoxic populations (CD8^+^ T-cells and γδ T-cells), exercise resulted in marked increases (mean fluorescent intensity (MFI) and percentage) in the inhibitory receptors PD-1 (*p* = 0.0089, *p* = 0.0301, respectively) and TIGIT (*p* = 0.0001, *p* = 0.0181, respectively), whereas expression on NK-cells remained unchanged (Figure 1B,C; Supplemental Figure S1). Conversely, exercise resulted in significant decreases in LAG-3 and TIM-3 expression and intensity on γδ T-cells (*p* = 0.0166, *p* = 0.0147, respectively). In NK-cells, only LAG-3 expression and MFI significantly decreased (*p* = 0.0118) (Figure 1D,E; Supplemental Figure S1) while expression of both markers on CD8^+^ T-cells remained unaltered by the acute exercise bout (Figure 1D,E).

Given the consistent exercise-induced elevation of TIGIT across T-cell subsets, we next examined whether TIGIT blockade could enhance PBMC cytotoxicity against leukemia cells. In a 24 h cytotoxicity assay (n = 9), TIGIT inhibition significantly increased lysis of K562 targets in both rest (*p* = 0.0354) and exercise PBMC conditions (*p* = 0.0091) (Figure 1F). Exercise PBMCs exhibited higher baseline cytotoxicity than rest PBMCs (*p* = 0.0306, 73% vs. 51%), and TIGIT blockade further augmented this effect, producing the greatest tumor lysis in the exercise + anti-TIGIT condition (EX + anti-TIGIT vs. REST *p* = 0.0020, 86% vs. REST + anti-TIGIT 58%).

### 3.2. Immune Checkpoint Ligand Expression Is Retained in Exercise-Expanded γδ T-Cells and TIGIT Blockade Enhances In Vitro Cytotoxic Function Against CML

**Figure 2:**
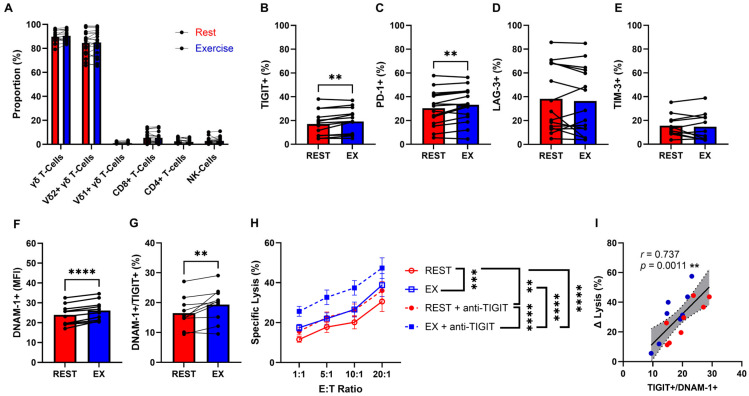
Exercise-expanded γδ T-cells preserve immune checkpoint ligand expression and exhibit enhanced in vitro cytotoxic function against CML with TIGIT blockade. (**A**) The cellular composition of expanded γδ T-cell products on day +14 of expansion. (**B**–**E**) The percentage of immune checkpoints among rest (REST)- and exercise (EX)-expanded γδ T-cells. (**F**) Mean fluorescent intensity of activating receptor DNAM-1 on REST- and EX-expanded γδ T-cells. (**G**) Co-expression of TIGIT and DNAM-1 on REST- and EX-expanded γδ T-cells. (**H**) The in vitro anti-tumor activity of REST- and EX-expanded γδ T-cells, with or without TIGIT-blockade (anti-TIGIT), against K562, assessed at 4-h via BLI. (**I**) Correlation between the change in cytolysis (ΔLysis) and γδ T-cells coexpressing TIGIT^+^ and DNAM1^+^; blue dots represent exercise-specific data points, red dots represent rest-specific data points. Data are represented as mean ± SEM; **, *p* < 0.01; ***, *p* < 0.001; ****, *p* < 0.0001 (**B**–**I**); two-tailed paired *t*-tests were used for statistical analyses (**B**–**G**). Repeated measures two-way ANOVA or LMM with Tukey’s *post hoc* test (**H**). Simple linear regression was used to measure ΔLysis in coexpressed TIGIT^+^/DNAM1^+^ γδ T-cells (**I**).

As described in our previous work [18], γδ T-cells expanded ex vivo for 14 days from rest- or exercise-derived PBMCs maintain a robust proliferative capacity and in vitro cytotoxic profile against numerous tumor lines (K562, Daudi, U266, 221.AEH, 721.221). In this study, after 14 days of expansion the final ACT product remained highly pure (>90% γδ T-cells) (Figure 2A), regardless of if the product was derived from rest- or -exercise PBMCs. These expanded γδ T-cells were phenotyped for expression and intensity (MFI) of immune checkpoint ligands, TIGIT, PD-1, TIM-3, and LAG-3 (Figure 2B–E; Supplemental Figure S2). Exercise-associated patterns of immune receptor modulation observed in γδ T-cells at the PBMC level were largely preserved after expansion. Specifically, surface expression and MFI of TIGIT (*p* = 0.0026) and PD-1 (*p* = 0.0068) were significantly elevated in the exercise-expanded γδ T-cells (Figure 2B,C; Supplemental Figure S2). However, there were no significant differences in expression of LAG-3 and TIM-3 (Figure 2D,E; Supplemental Figure S2). Importantly, exercise-expanded γδ T-cells exhibited increased DNAM-1 expression (Figure 2F) and higher proportion of TIGIT^+^/DNAM-1^+^ co-expression (Figure 2G), identifying a sub-population of cytotoxic γδ T-cell restricted by inhibitory signals.

Based on our previous work [18], exercise-expanded γδ T-cells enhanced cytolysis of K562 require DNAM-1/PVR interaction, therefore, we investigated whether the combination of TIGIT-blockade, with exercise-expanded γδ T-cells could enhance anti-leukemic activity against K562 cells in vitro (Figure 2H). From our in vitro cytotoxicity assays, we confirmed that the improved cytotoxic effect of exercise-expanded γδ T-cells alone, reported by Baker et al., was maintained (~7%). Rest- and exercise-expanded γδ T-cells in the presence of TIGIT blockade exhibited increased anti-leukemic effect on K562 cells, demonstrating specific lysis (20:1 E:T) of 36% and 47.3% (*p* < 0.0001), respectively. While exercise-expanded γδ T-cells with (47.3%) and without (38.8%) TIGIT blockade, maintained the greatest cytolysis (*p* < 0.0001). Notably, the magnitude of γδ T-cell–mediated cytotoxicity following TIGIT blockade positively correlated with the proportion of TIGIT^+^/DNAM-1^+^ co-expressing γδ T-cells (Figure 2I), suggesting that enhanced lysis is associated with relief of inhibitory signaling within this effector subset. These results suggest that TIGIT-blockade may significantly augment exercise-expanded γδ T-cell lysis of CML cells, underlining the combinations’ potential as a therapeutic strategy for further investigation among alternative hematological malignancies.

### 3.3. TIGIT Blockade Trends Toward Improved Tumor Suppression with Exercise-Expanded γδ T-Cells In Vivo

**Figure 3:**
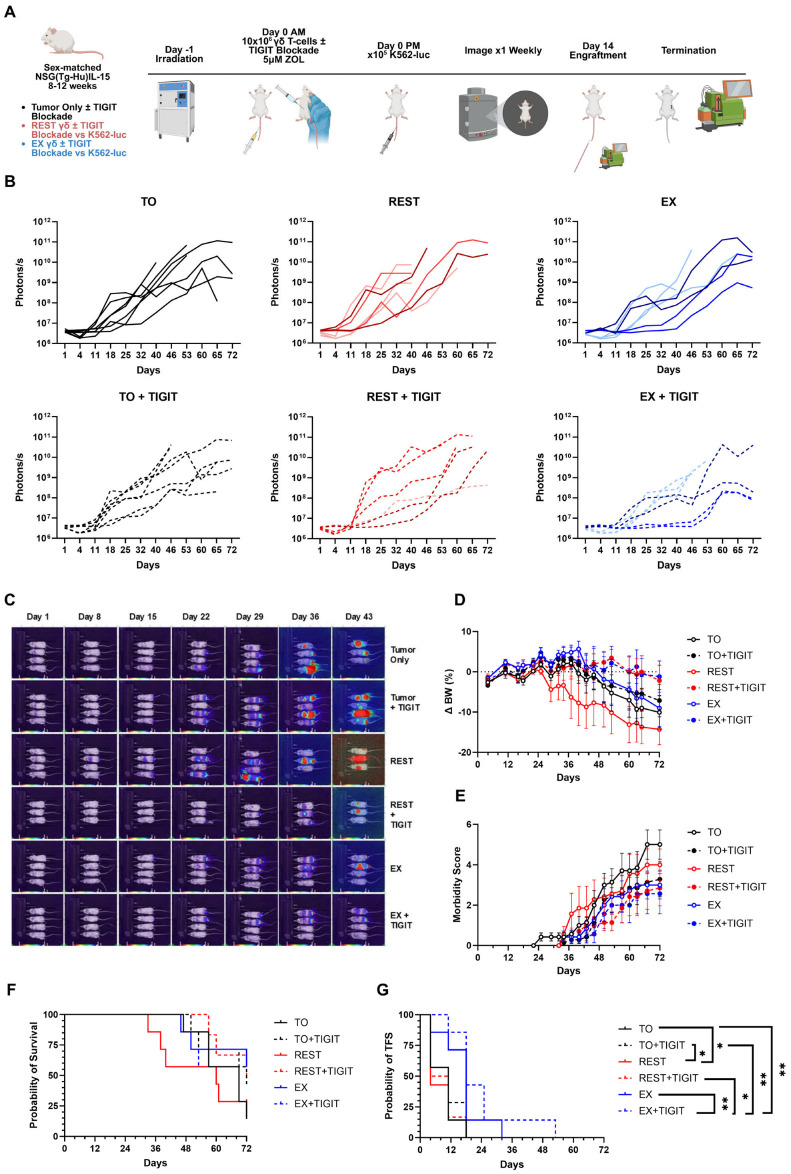
Exercise-expanded γδ T-cells exhibit a trend toward improved tumor suppression in vivo following TIGIT blockade. (**A**) Schematic of the experimental design for in vivo experiment to determine the anti-leukemia effect of exercise (EX)-expanded γδ T-cells with TIGIT blockade (+TIGIT). Briefly, NSG-IL15 mice were injected in the tail vein on Day 0 with REST or EX-expanded γδ T-cells (10 × 10^6^) treated with a TIGIT blockade (+TIGIT) or untreated (IgG Control), and challenged 4 h later with 5 × 10^5^ luciferase tagged human CML cells (K562-luc). (**B**) Individual, within-group mouse bioluminescence. Line shading defines individual donors (n = 3) in treatment condition groups. (**C**) Representative BLI of leukemia-bearing mice that received (from top to bottom) Tumor only (n = 7), Tumor + TIGIT blockade (n = 7), REST-expanded γδ T-cells (n = 7), REST-expanded γδ T-cells + TIGIT blockade (n = 6), EX-expanded γδ T-cells (n = 7), and EX-expanded γδ T-cells + TIGIT blockade (n = 7). BLI intensity on a scale from low (purple) to high (red). (**D**–**G**) Change in BW, morbidity score, probability of tumor free survival (TFS), and overall probability of survival after injection of expanded γδ T-cells with or without TIGIT blockade. Engraftment of γδ T-cells was equal among mice. Data are represented as mean ± SEM; *, *p* < 0.05; **, *p* < 0.01; repeated measures two-way ANOVA with Bonferroni post hoc test. Kaplan–Meier curves were generated to assess overall and tumor free survival.

We next evaluated whether TIGIT blockade could augment the anti-leukemic activity of rest- and exercise-expanded γδ T-cells in vivo. NSG-Tg(Hu-IL15) mice received a single prophylactic intravenous dose of expanded γδ T-cells that had been pre-incubated with either an anti-TIGIT antibody (10 μg/g BW) or IgG isotype control. Four hours later, mice were challenged intravenously with 5 × 10^5^ K562-luc leukemia cells and monitored longitudinally for tumor burden, tumor-free survival, and overall survival (Figure 3A). Tumor progression was assessed weekly by bioluminescent imaging (Figure 3B,C), and body weight and morbidity were evaluated twice weekly (Figure 3D,E). Untreated mice displayed a median tumor-free survival of 13 days, whereas mice receiving rest- or exercise-expanded γδ T-cells exhibited extended tumor-free survival of 21 and 26 days, respectively. γδ T-cell treatment alone suppressed tumor growth until approximately day 40, while the addition of TIGIT blockade further prolonged tumor control to day 47 (Figure 3B,C,G). Despite these improvements in tumor suppression and tumor-free survival (Figure 3G), no significant differences in overall survival were observed among treatment groups (Figure 3F). These findings suggest that TIGIT blockade provides an additive protective effect when combined with γδ T-cell therapy, slowing leukemic progression more effectively than γδ T-cells alone, and may be cemented further by repeated combination treatments, similar to clinical settings.

## 4. Discussion

This study provides novel insights into how acute exercise can prime DLIs and expanded γδ T-cells for enhanced anti-leukemic activity when combined with immune checkpoint inhibition. We found that acute exercise significantly altered the immune checkpoint landscape of circulating PBMCs, most notably increasing TIGIT and PD-1 expression on CD8^+^ and γδ T-cells, and cytotoxicity against K562 leukemia cells, which was further enhanced by TIGIT blockade. Furthermore, γδ T-cells expanded from exercise-derived PBMCs retained elevated TIGIT and PD-1 expression and TIGIT blockade augmented the DNAM-1-dependent cytotoxicity in vitro. Finally, in vivo, combining TIGIT blockade with exercise-expanded γδ T-cells modestly improved tumor control and prolonged tumor-free survival without increasing GvHD. Together, these findings suggest that exercise-induced immune priming sensitizes γδ T-cells to checkpoint inhibition and that targeting TIGIT may help overcome key functional barriers in γδ T-cell–based immunotherapy for leukemia.

High expression of immune checkpoint receptors (PD-1, CTLA-4, LAG-3) is a well-established driver of lymphocyte inactivation, exhaustion, and anergy [50]. This exhausted phenotype impairs the release of key cytotoxic effectors such as TNFα, IFN-γ, and granzyme within the TME [51]. However, the profile of immune checkpoint expression on lymphocytes following acute exercise remains poorly characterized, with limited studies reporting marginal increases in circulating PD-1^+^/CD4^+^ and PD-1^+^/CD8^+^ T-cells after an acute bout of exercise [52,53,54]. Our findings extended this knowledge by demonstrating that acute exercise reshapes inhibitory receptor expression, selectively increasing PD-1 and TIGIT expression on CD8^+^ T-cells and γδ^+^ T-cells. In addition, we observed a favorable decrease in LAG-3 and TIM-3 expression on γδ T-cells and NK-cells (LAG-3 only) mobilized with exercise. These changes may reflect mobilization of lymphocyte subsets with distinct checkpoint profiles, transient alterations in receptor regulation, or early activation signatures. Importantly, the acute reduction of TIM-3 and LAG-3 on γδ T-cells suggests that exercise does not uniformly increase inhibitory pressure across all pathways but instead produces a nuanced, receptor-specific modulation. While we observed changes in checkpoint inhibitor expression among cytotoxic lymphocytes, exercise induces a complex, multifactorial immune response and it is possible that changes in inhibitory receptor expression reflect secondary adaptations within an already activated effector cell population rather than a primary mechanistic driver. Future studies should explore the neuroendocrine signaling and inflammatory mediators that may contribute to the observed functional effects. Regardless, this raises the possibility that exercise induces a poised effector state, characterized by enhanced cytotoxic potential yet vulnerable to TIGIT-mediated suppression, which may be strategically targeted by checkpoint inhibition.

The DNAM-1/PVR axis is central to effector cell recognition and killing of leukemia cells, as DNAM-1 is a key activating receptor required for efficient targeting of hematological malignancies and its ligand PVR is frequently overexpressed on leukemia cells [55,56]. Conversely, TIGIT outcompetes DNAM-1 for PVR engagement, suppressing downstream signaling and effector cell mediated cytotoxicity [57]. We have previously demonstrated that exercise-expanded γδ T-cells rely on the DNAM-1/PVR axis to recognize and lyse leukemic cells [18]. Specifically, DNAM-1 expression was upregulated on exercise-expanded γδ T-cells and blockade of DNAM-1 or PVR/Nectin-2 abrogated the cytotoxic efficacy of γδ T-cells, however, TIGIT-mediated disruption of this axis was never investigated. Here we observed that exercise-expanded γδ T-cells maintained an elevated expression of TIGIT and PD-1, as observed in the exercise mobilized PBMCs. Importantly, we identified a subset of DNAM-1^+^/TIGIT^+^ γδ T-cells that exhibit strong cytotoxic potential when TIGIT is blocked. This shift in TIGIT is consistent across PBMCs and expanded γδ T-cell products, highlighting it as a dominant inhibitory checkpoint in exercise-primed γδ T-cell biology and TIGIT blockade likely confers DNAM-1 access to PVR, enabling activation, degranulation, and cytotoxicity. These findings represent one of the first demonstrations of this mechanism in expanded γδ T-cells following acute exercise and future work should investigate TIGIT expression and interaction, as well as a combination treatment with other exercise-mobilized products, such as NK-cells, CD8^+^ T-cells, and CD4^+^ T-cells.

Our group has previously demonstrated the expansion capabilities of exercise-mobilized PBMCs and exercise-expanded γδ T-cells, as well as their significantly increased anti-tumor capabilities against multiple hematological cell lines (K562, U266, Daudi) in vitro [18]. To better understand the implications of these phenotypic shifts of TIGIT on exercised lymphocytes, we replicated an established model of DLI versus K562 [18,43]. We reproduced the improved anti-leukemic effects of exercise-derived DLIs against a K562 target alone, and discovered further cytotoxicity was reported when a TIGIT blockade was added to both rest and exercise conditions. Indeed, acute exercise significantly alters the intracellular composition of immune checkpoint inhibitors in leukocyte subsets, particularly TIGIT and PD-1 in CD8^+^ and γδ^+^ T-cells. Notably, exercise and TIGIT blockade acted additively in PBMC cultures, suggesting that exercise mobilizes effector populations predisposed to respond to checkpoint inhibition. Several studies have documented the increased transcriptional and phenotypic diversity of exercise-mobilized lymphocytes, including elevated granzyme expression, cytotoxic signaling, and proliferative capacity [43,58,59,60]. Future studies should complete a comprehensive insight into effects across other CML lines and further replication of the methodology utilized would be required to make a global conclusion that represent potentially more aggressive and treatment-resistant subtypes. Given these findings, we can proport that ICI, particularly via TIGIT, is effective in restoring effector activation and anti-tumoral activity against a CML model, K562, and may be required to fully unlock the cytotoxic potential of these cells in leukemic environments.

Given the challenges facing adoptive cellular therapies, and the growing interest in combining immune checkpoint inhibition with allogeneic products, this study focused on DLI, DLI-X, and γδ T-cells. For decades, γδ T-cells have been established as a safe and feasible platform for cancer immunotherapy [61,62,63]. While clinical efficacy in hematological malignancies has historically been modest, recent research suggests that acute exercise reprograms these cells, creating a more robust and persistent “off-the-shelf” product [18,36,38]. However, the specific impact of exercise on the checkpoint profile of this subset remains under-characterized. The clinical implications of these findings are substantial, as exercise-derived effector lymphocytes, including γδ T-cells, may represent superior ACT products due to their enhanced cytotoxicity, proliferative capacity, and transcriptomic diversity. Pairing such cells with TIGIT inhibitors, several of which are under active clinical investigation, could offer a non-toxic, biologically rational strategy to improve leukemia immunotherapy. TIGIT expression on γδ T-cells in leukemia patients has been correlated with poor outcomes and dysfunctional immune activity; thus, targeted TIGIT blockade may restore activity in patient-derived cells as well. Moreover, the safety profile observed in this study supports continued exploration of TIGIT inhibitors in combination with adoptive cellular therapies. The in vivo results, although modest in magnitude, provide important translational insight as TIGIT blockade combined with exercise-expanded γδ T-cells delayed leukemic progression and extended tumor-free survival more effectively than γδ T-cells alone, when administered prophylactically. However, the use of prophylactic injection limits relatability to clinical practice in this tumor model and may affect tumor control and effector cell persistence, compared to repeated treatment [64]. In our study, the single injection of expanded γδ T-cells and the short timeframe from effector combination (γδ T-cell + TIGIT blockade) treatment to introducing tumor potentially limited the overall response in vivo and future work should optimize the treatment timing and explore repeated injection of expanded γδ T-cells to mimic clinical models. Although overall survival was unchanged and despite the room for optimization, the enhanced tumor control and maintained safety profile observed here supports continued investigation of TIGIT-targeted strategies in combination with exercise-expanded γδ T-cells within the leukemic tumor microenvironment.

Despite substantial advances in CML treatment, including tyrosine kinase inhibitors, chemotherapy, and hypomethylating agents, treatment resistance, residual disease, and immune dysfunction remain significant clinical challenges, with each modality introducing safety risks and treatment-related toxicities [65,66]. As durable, treatment-free remission is achieved in only a subset of patients [67,68], there is a clear need to enhance GvL efficacy through novel immunotherapeutic strategies. Although immune checkpoint inhibition carries potential toxicity risks [69,70], it remains a promising combinational strategy to enhance allogeneic effector cell function by relieving inhibitory signaling within the leukemic microenvironment. Accordingly, numerous preclinical studies are actively evaluating mono- and combination checkpoint blockade approaches targeting inhibitory receptor–ligand axes (PD-1/PD-L1, CTLA-4/CD80 and CD86, TIM-3/Galectin9, and TIGIT-CD155/CD112) in leukemia models [16,71,72,73,74]. Importantly, we observed elevated expression of both PD-1 and TIGIT on T-cell populations and given the enhanced in vitro cytotoxicity and modest in vivo efficacy achieved through TIGIT blockade of exercise-expanded DLI and γδ T-cell products, these findings provide a strong rationale for investigating dual checkpoint inhibition strategies. Indeed, checkpoint inhibitor expression changes observed as a result of acute exercise may represent a secondary adaptation among lymphocytes, following the primary exercise-induced changes–transcriptomic and phenotypic-among the immune millieu associated with mechanistic driving of enhanced cytotoxicity [18]. Further understanding of the sequence in which immune checkpoint inhibition elicits a potentiation of exercise-primed-T-cell cytotoxicity will be necessary to define the interaction and the in-depth contributions of blockade.

Co-targeting PD-1 or PD-L1 alongside TIGIT may further bolster preclinical and in vivo responses by relieving complementary inhibitory pathways. Both PD-1 and TIGIT transmit distinct inhibitory signals that converge to suppress innate and adaptive immune effector functions, including CD226 (DNAM-1)-mediated co-stimulation, cytokine production (IFN-γ, TNF-α), granzyme B release, and NK-cell-mediated cytotoxicity [75,76,77]. Consistent with this model, prior studies demonstrate that dual blockade of TIGIT and PD-1 synergistically restores CD226 signaling and enhances the expansion, functional fitness, and antitumor efficacy of tumor-reactive T-cells beyond that achieved by inhibition of either pathway alone [76,78]. Moreover, blockade of the PD-1/PD-L1 and CTLA-4/CD80/CD86 axes, either alone or in combination with established AML therapies such as decitabine, venetoclax, azacitidine, or post-transplant cyclophosphamide, has produced encouraging, though generally modest, responses in preclinical leukemia models.

In the setting of allogeneic hematopoietic cell transplantation (alloHCT), a key challenge remains optimizing GvL activity while limiting GvHD, as well as defining the optimal timing of checkpoint intervention. Although immune checkpoint inhibitor-associated GvHD has been reported both pre- and post-alloHCT with considerable variability [31], PD-1/PD-L1 blockade has shown the most consistent signals of clinical activity across studies. While outcomes to date remain mixed, the dual targeting of TIGIT and PD-1/PD-L1 represents a mechanistically compelling strategy with the potential to enhance cytotoxic effector function in leukemia and warrants further investigation.

## 5. Conclusions

In summary, our results demonstrate that exercise mobilized lymphocytes and exercise-expanded γδ T-cells have a checkpoint receptor profile that sensitizes them to TIGIT blockade. TIGIT inhibition reinvigorates DNAM-1-dependent killing pathways, enhancing both PBMC and exercise-expanded γδ T-cell cytotoxicity against leukemia. Moreover, combining exercise-expanded γδ T-cells with TIGIT blockade, in vivo, modestly delays tumor progression while maintaining safety. Together, these findings establish mechanistic foundation and translational rationale for integrating exercise-derived cell therapies with checkpoint inhibitors to advance DLI and γδ T-cell-based immunotherapy.

## Figures and Tables

**Table 1:** Total leukocyte subset counts (cells/μL) in peripheral blood at rest and during exercise. Main effect reported from paired *t*-tests. Data are mean ± SD, n = 26. Significance is indicated by ** *p* < 0.01; ***, *p* < 0.001; ****, *p* < 0.0001.

Leukocyte Subset	Rest (Cells/µL ± SD)	Exercise (Cells/µL ± SD)	Effect
Lymphocytes	1911.32 ± 537	3670.96 ± 992.52	*p* < 0.0001 ****
Monocytes	349.08 ± 113.96	734.93 ± 241.04	*p* < 0.0001 ****
CD3+ T-cells	1175.50 ± 381.12	1774.32 ± 609.52	*p* < 0.0001 ****
CD4+ T-cells	612.73 ± 215.56	811.32 ± 290.63	*p* < 0.0001 ****
CD8+ T-cells	389.89 ± 196.06	669.30 ± 342.47	*p* < 0.0001 ****
CD4+/CD8+ T-cells	40.28 ± 25.02	51.33 ± 32.31	*p* = 0.0052 **
CD4-/CD8- T-cells	115.55 ± 71.82	214.26 ± 130.74	*p* < 0.0001 ****
γδ T-cells	112.71 ± 83.86	193.82 ± 81.63	*p* = 0.0004 ***
NK T-cells	123.01 ± 95.18	255.35 ± 192.62	*p* = 0.0001 ***
NK Cells	365.61 ± 185.68	1275.01 ± 446.71	*p* < 0.0001 ****
B-cells	254.51 ± 161.52	360.84 ± 205.33	*p* < 0.0001 ****

## Data Availability

The data presented in this study are available upon request from the corresponding author.

## Supplementary Materials

The following supporting information can be downloaded at: https://www.mdpi.com/article/10.3390/cancers18050797/s1. Table S1. Flow cytometry phenotyping panels and marker combinations used to identify lymphocyte and monocyte lineages, define γδ T-cell phenotypes, and quantify immune checkpoint receptor expression on PBMCs and γδ T cells, respectively. Figure S1. CPI MFI’s. Figure S2. Gamma Delta Post-Expansion CPI MFI’s.

## Abbreviations

ACT	Adoptive cellular therapy
ACSM	American College of Sports Medicine
AHA	American Heart Association
alloHCT	Allogeneic hematopoietic cell therapy
AML	Acute myeloid leukemia
ANOVA	Analysis of variance
BLI	Bioluminescent imaging
BMI	Body mass index
BP	Blood pressure
BPM	Beats per minute
CD	Cluster of differentiation
CML	Chronic myeloid leukemia
CTLA-4	Cytotoxic T-lymphocyte-associated protein 4
DLI	Donor lymphocyte infusion
DLI-X	Exercised donor lymphocyte infusion
DNAM-1	DNAX accessory molecule-1
EX	Exercise
E:T	Effector-to-target
GvHD	Graft-versus-host disease
GvL	Graft-versus-Leukemia
Hr	Hour
ICI	Immune checkpoint inhibitor
IFN	Interferon
Kg	Kilogram
Kg/m^2^	Kilogram per meter squared
Luc	Luciferin/Luciferase
MFI	Mean fluorescent intensity
MHC	Major histocompatibility complex
MICA/MICB	Major histocompatibility complex class I polypeptide-related sequence A/B
mmHg	Millimeters of Mercury
mL/dL	Milliliter per deciliter
mL/min/kg	Milliliter per minute per kilogram
NK	Natural killer
PBMC	Peripheral blood mononuclear cells
PD-1	Programmed death protein-1
PDL-1	Programmed death ligand protein-1
PVR	Poliovirus receptor
RHR	Resting heart rate
RPE	Rate of perceived exertion
RPM	Revolutions per minute
SD	Standard deviation
SEM	Standard error of the mean
TIGIT	T-cell immunoreceptor with Ig and ITIM domains
TIM-3	T-cell immunoglobulin and mucin domain-containing molecule 3
TFS	Tumor-free survival
TKI	Tyrosine kinase inhibitor
TME	Tumor microenvironment
TNF	Tumor necrosis factor
TRAIL	Tumor necrosis factor-related apoptosis-inducing ligand
ULBP	Ubiquitin like protein
$\dot{V}$O_2_max	Maximal oxygen uptake
γδ	Gamma Delta
ZOL	Zoledronate/Zoledronic acid
