# Supplementary material for: TIGIT Blockade Potentiates the Anti-Leukemic Activity of Exercise-Mobilized Donor Lymphocytes and Expanded γδ T-Cells
Source: Cancers (Basel). 2026 Feb 28;18(5):797. doi: 10.3390/cancers18050797 (PMC12984231; doi:10.3390/cancers18050797)
Supplement: Supplementary file 1 [file cancers-18-00797-s001.zip › cancers-4140204-supplementary.pdf]

Table S1. Flow cytometry phenotyping panels and marker combinations with clones used to identify lymphocyte and monocyte lineages, define γδ T-cell phenotypes, and quantify immune checkpoint receptor expression on PBMCs and γδ T cells, respectively.

Channel	V1	V2	B1	B2	B3	B5	B6	R1	R3
Lymphocyte/ Monocyte Lineage Markers*	CD8 (BW135/80)	CD3 (REA613)	CD14 (REA599)	CD4 (REA623)	CD45 (REA747)	TCR-γδ (11F2)	CD20 (LT20)	CD16 (REA423)	CD56 (REA196)
γδ T-cell Phenotype	CD8 (BW135/80)	CD3 (REA613)	TCR-Vδ2 (REA771)	CD4 (REA623)	CD45 (REA747)	TCR-γδ (11F2)	TCR-αβ (REA652)	CD16 (REA423)	TCR-Vδ1 (REA173)
Checkpoint Inhibitor Phenotype (on PBMCs)	TIGIT† (A1513G)	CD8 (BW135/80)	CD3 (REA613)	TIM-3‡ (F38-2E2)	CD45 (REA747)	TCR-γδ (11F2)	LAG-3‡ (3DS223H)	PD-1 (REA1165)	CD56 (REA196)
Checkpoint Inhibitor Phenotype (on γδ T-cells)	TIGIT† (A1513G)	CD3 (REA613)	TCR-Vδ2 (REA771)	TIM-3‡ (F38-2E2)	CD45 (REA747)	TCR-γδ (11F2)	LAG-3‡ (3DS223H)	PD-1 (REA1165)	CD56 (REA196)

V, B, and R denote violet, blue and red laser channels, respectively; numbers indicate individual detector channels corresponding to specific fluorochromes as configured on the flow cytometer.

* asterisk represents samples were analyzed in whole blood sample.

All antibodies were acquired from Miltenyi Biotec unless otherwise denoted by †BioLegend, or ‡Invitrogen.
