# Supplementary figures and images for: TIGIT Blockade Potentiates the Anti-Leukemic Activity of Exercise-Mobilized Donor Lymphocytes and Expanded γδ T-Cells
Source: Cancers (Basel). 2026 Feb 28;18(5):797. doi: 10.3390/cancers18050797 (PMC12984231; doi:10.3390/cancers18050797)

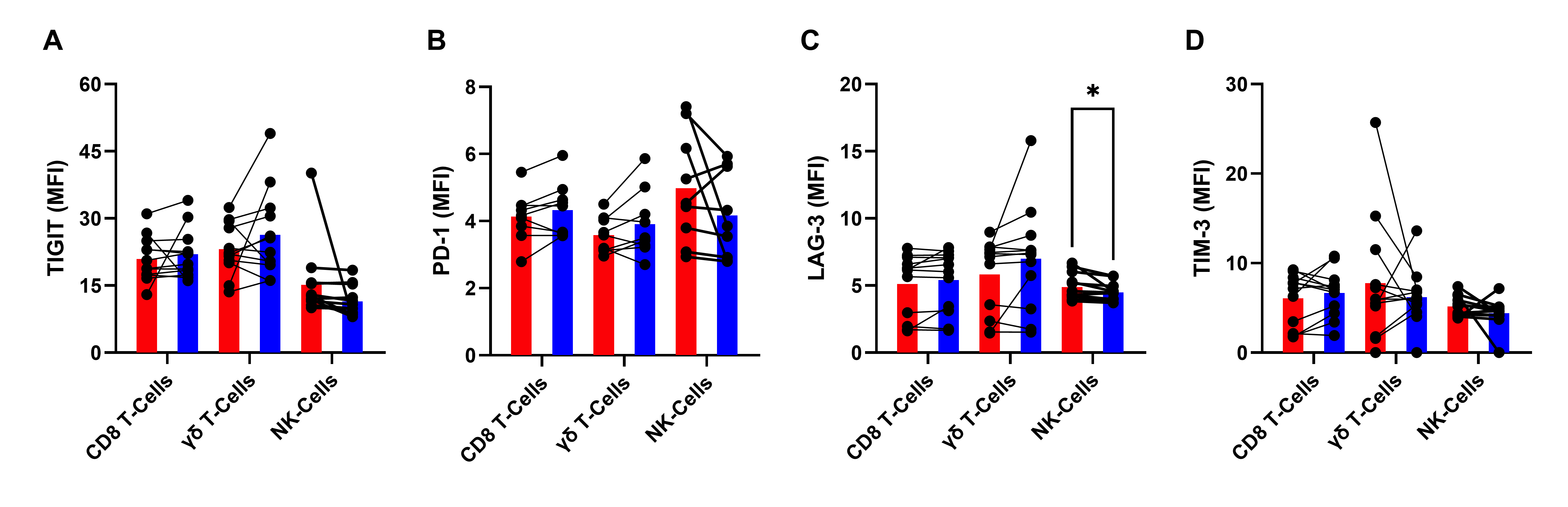

Supplement: Supplementary file 1 [file cancers-18-00797-s001.zip › Supplemental Figure S1 CPI MFI's.tif]

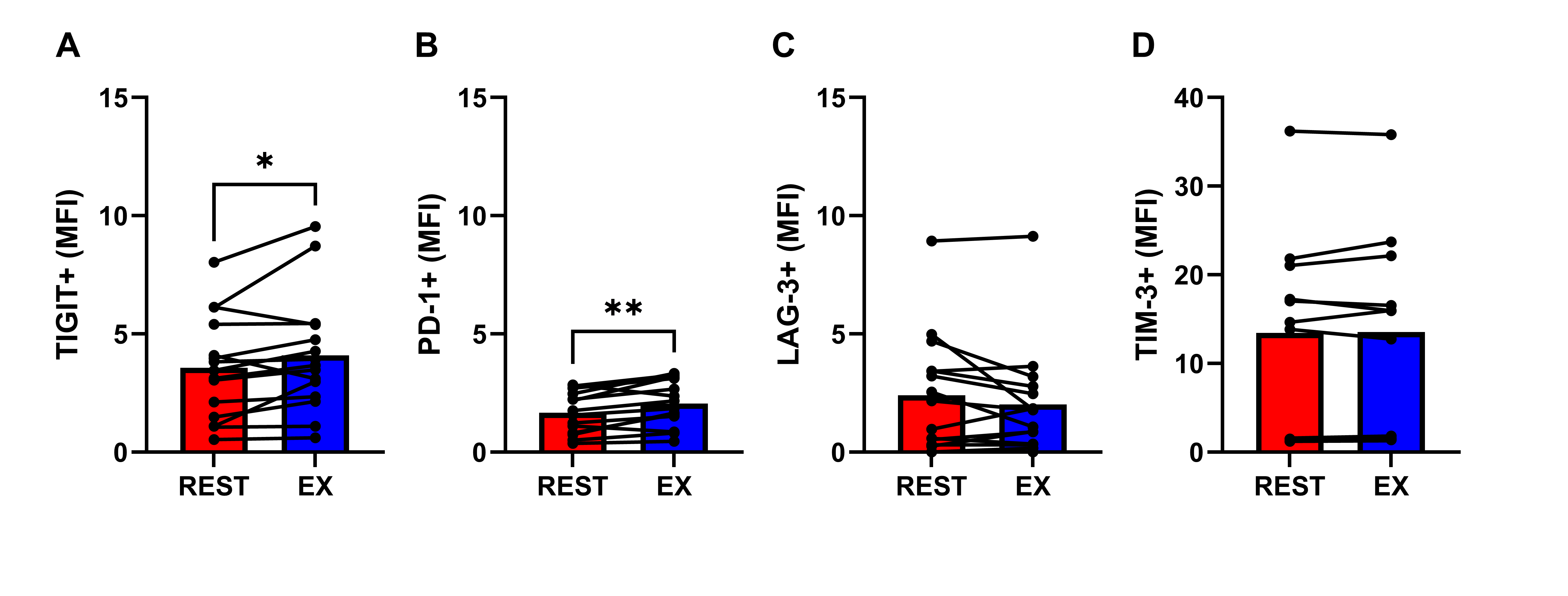

Supplement: Supplementary file 1 [file cancers-18-00797-s001.zip › Supplemental Figure S2 Gamma Delta Post-Expansion CPI MFI's.tif]
